# Enhancing Therapeutic Processes in Videoconferencing Psychotherapy: Interview Study of Psychologists’ Technological Perspective

**DOI:** 10.2196/40542

**Published:** 2023-03-16

**Authors:** Francesco Cataldo, Antonette Mendoza, Shanton Chang, George Buchanan, Nicholas T Van Dam

**Affiliations:** 1 School of Computing and Information Systems Faculty of Engineering and Information Technology University of Melbourne Melbourne Australia; 2 School of Psychological Sciences Faculty of Medicine, Dentistry and Health Sciences University of Melbourne Melbourne Australia

**Keywords:** videoconference psychotherapy, therapeutic relationship, therapeutic alliance, telehealth, technology, therapeutic processes

## Abstract

**Background:**

The COVID-19 pandemic caused a surge in the use of telehealth platforms. Psychologists have shifted from face-to-face sessions to videoconference sessions. Therefore, essential information that is easily obtainable via in-person sessions may be missing. Consequently, therapeutic work could be compromised.

**Objective:**

This study aimed to explore the videoconference psychotherapy (VCP) experiences of psychologists around the world. Furthermore, we aimed to identify technological features that may enhance psychologists’ therapeutic work through augmented VCP.

**Methods:**

In total, 17 psychologists across the world (n=7, 41% from Australia; n=1, 6% from England; n=5, 29% from Italy; n=1, 6% from Mexico; n=1, 6% from Spain; and n=2, 12% from the United States) were interviewed. We used thematic analysis to examine the data collected from a sample of 17 psychologists. We applied the Chaos Theory to interpret the system dynamics and collected details about the challenges posed by VCP. For collecting further information about the technology and processes involved, we relied on the Input-Process-Output (IPO) model.

**Results:**

The analysis resulted in the generation of 9 themes (input themes: psychologists’ attitude, trust-reinforcing features, reducing cognitive load, enhancing emotional communication, and engaging features between psychologists and patients; process themes: building and reinforcing trust, decreasing cognitive load, enhancing emotional communication, and increasing psychologist-patient engagement) and 19 subthemes. Psychologists found new strategies to deal with VCP limitations but also reported the need for more technical control to facilitate therapeutic processes. The suggested technologies (eye contact functionality, emergency call functionality, screen control functionality, interactive interface with other apps and software, and zooming in and out functionality) could enhance the presence and dynamic nature of the therapeutic relationship.

**Conclusions:**

Psychologists expressed a desire for enhanced control of VCP sessions. Psychologists reported a decreased sense of control within the therapeutic relationship owing to the influence of the VCP system. Great control of the VCP system could better approximate the critical elements of in-person psychotherapy (eg, observation of body language). To facilitate improved control, psychologists would like technology to implement features such as improved eye contact, better screen control, emergency call functionality, ability to zoom in and out, and an interactive interface to communicate with other apps. These results contribute to the general perception of the computer as an actual part of the VCP process. Thus, the computer plays a key role in the communication, rather than remaining as a technical medium. By adopting the IPO model in the VCP environment (VCP-IPO model), the relationship experience may help psychologists have more control in their VCP sessions.

## Introduction

### Background

Since the onset of the COVID-19 outbreak, there has been a dramatic increase in telehealth use [1]. Most clinicians, including psychologists, rapidly shifted from the traditional face-to-face (FTF) approach to web-based videoconference psychotherapy (VCP) [1]. Psychologists began to rely exclusively on video and audio channels to support their patients during the pandemic [2]. Although the COVID-19 pandemic encouraged psychologists to undertake VCP as a temporary alternative to FTF treatment, the uptake of such approaches was already rising [3], especially in remote areas where access to health care services is limited [4].

The recent pandemic-related wide-scale shift to telehealth has altered psychologists’ traditional modus operandi, thus compromising their therapeutic work. According to Bowlby [5], “the psychologist strives to be reliable, attentive, and sympathetically responsive to his patient’s exploration, and so far as he can, to see and feel the world through his patient’s eyes, namely to be empathic.” Regardless of the therapeutic approach used, psychologists facilitate change among patients, leading to few *dysfunctional schemas* and widening patients’ perspectives [6]. Most clinicians agree on core nonspecific factors essential to therapeutic outcomes, and according to Wampold [6], these factors include the engagement of psychologist and patient in the work of therapy and patient’s expectancy regarding the process and the results of the therapy. However, these components hinge upon the therapeutic relationship (TR; ie, relationship between client and psychologist).

TR is “the personal relationship between psychologist and patient marked by the extent to which each is genuine with the other and perceives/experiences the other in ways that benefit the other” [7]. TR refers to an authentic rapport implying the development of trust and empathy [8,9].

In TR, the interaction is strictly confidential (with some exceptions) and possible revelations hypothetically should not affect the bond [6]. TR also includes the establishment of the therapeutic alliance (TA), which refers to psychologist-client connection and cooperation for the achievement of the therapy goals [8,10,11].

Hatcher and Barends [12] explained the alliance as “the degree to which the therapy dyad is engaged in collaborative, purposive work*.”* Given the critical role of these relational features (ie, TA and TR), it is essential to explore how they are affected by telehealth platforms. Moreover, these platforms need to be optimized to help psychologists conduct their work comparably with FTF treatment.

Pilot studies demonstrate that FTF therapy and VCP enable similar levels of TA, and VCP achievement is more affected by the personality traits of psychologists and patients than by technology interference [13]. Moreover, literature reviews on VCP underline substantial reduction of symptoms in conditions such as depression, eating disorders, and posttraumatic stress disorder [14-17]. However, these studies do not juxtapose videoconference and FTF processes.

Literature contrasts patients’ and psychologists’ satisfaction with the separate settings that VCP imposes [1,18]. According to studies conducted during the COVID-19 pandemic, patients treated by VCP tend to not perceive the psychologist’s presence as intimidating as in FTF therapy, and they interact more spontaneously with the psychologist; in contrast, psychologists evaluate VCP as difficult in perceiving patients’ physical and emotional signals [1,19-22]. Psychologists have felt uncertain about VCP adoption during the entire duration of the pandemic [23,24], but at present, they appear more open to the use of VCP than before the pandemic [25].

Several critical questions arise when considering the return to normal life post–COVID-19: will psychologists return to FTF therapy or continue to work using VCP in some circumstances? Given the difficulties involved in predicting future trends, it is important to understand the role and impact of VCP on mental health services.

The lack of presence, fragility of trust, limited ability to make eye contact, and partial body language [26] all negatively affect video communication. Moreover, these elements contribute to the development of TA and TR. Hence, declines in these elements may undermine both.

Bekes et al [27] compared the previous and current experiences of 190 psychoanalytic psychologists transitioning to web-based therapy. The results of the survey indicated that although they had technical and relational issues with their patients, they continued to be resilient, emotional, and trustworthy. Despite these positives, most believed that web-based therapy was not as efficient as FTF therapy. Psychologists were especially concerned by the inability to observe body language, limited ability to observe facial expressions, and limitations in detection of vocal tone [26,28]. Therefore, VCP threatens to jeopardize the establishment of the psychologist-patient relationship owing to the absence of physical presence—a critical element in developing therapeutic processes [29-31]. However, the literature on the topic draws more attention to psychologists’ and patients’ preferences, rather than exploring why psychologists feel that VCP is an impediment to the therapeutic process.

Despite the indication that the therapeutic process is potentially impaired by technology, the mechanism by which this occurs is unclear. The processes involved in VCP are part of a complex system governed by technology. This system affects the whole therapy process, and we believe that research should provide a definitive analysis of the multidynamic aspects involved in therapeutic communication and relationship. Thus, the guiding questions to be addressed in this paper are 2-fold:

1. What processes are involved in VCP from psychologists’ perspective?
2. What technological features support psychologists’ need to develop these processes?

To address these questions, we applied the Chaos Theory, which investigates how small alterations in a part of the system generate large alterations in the whole process. Furthermore, we also relied on the Input-Process-Output (IPO) model to collect further information about the technology and processes involved. The IPO model relies on the theory that a specific input initiates a process that produces an output [32].

This study aimed to analyze the processes involved in the VCP system and explore possible technological features that may improve and enhance psychologists’ VCP experience and therapeutic processes.

In the following section, we propose an overview of the elements that the literature highlights as critical: presence, trust, eye contact, and body language.

Then, we present the framework adopted to conduct our study. Subsequently, we present our methodology; results; and finally, the discussion and conclusion of our study.

### Presence

Presence is crucial for developing and building empathy and alliance and facilitating cognitive and emotional processes [9]. For a psychologist, presence is defined by the interaction with the environment [33]. Psychologists demonstrate their presence by interacting dynamically with the physical setting of the therapy room and connecting interpersonally with the client [9]. Psychologists and clients share the same environment and instant, which helps them bond; furthermore, the presence consents the development of TR [1,29-31].

Previous study [34] shows how embodied factors play a crucial role in therapeutic mechanisms [35,36]. In FTF therapy, the bodily experience of the psychologist and patient allows the mutual exchange of body and behavioral signals [37]. The psychologists and clients receive, process, and respond to these signals by coordinating movements and emotions based on their social interaction, that is, adjusting their tone of voice or posture to fit the social context [38]. This process, called *participatory sense making,* refers to how individuals interpret interactions within psychotherapy [34]. As a consequence, in FTF sessions, the physical presence and *intracorporal synergies* support the establishment of TA and therapeutic change mechanisms [39].

However, the literature highlights the difficulties in developing copresence by VCP (ie, the sensation of being included in a web-based place with other remote people) [40]. Physical presence provides the possibility to influence the environment [41] and generates a common ground that helps people to share experiences [42]. Hence, the complexity of producing copresence by VCP compromises the chances of interacting by exploiting common cues, and this may alter the achievement of communication and compromise the development of trust [43].

In the following section we illustrate the concept of trust applied to psychotherapy and VCP.

### Trust

Trust is defined as “a willingness to be vulnerable, based on positive expectations about the actions of others” [44]. Studies in computer-mediated communication highlight the fragility of trust among users [1,26,45], and scholars argue that the development of trust is influenced by proximity and hampered by sight restriction [46,47].

In psychotherapy, clients’ trust is nourished when the psychologists’ actions are perceived as positive for the therapeutical relationship and convey a sense of safety and protection [48]. According to Willis and Todorov [49], from the first moments of therapy, clients make a snap judgment about the psychologist and begin to consider trust. Therefore, therapy is precarious from the very initial moments of the first session, a point underscored by the fact that many clients quit therapy after the first session [50]. Hence, trust is a crucial element in therapy [1,26,48], and trust is established in a fundamentally different way via VCP relative to FTF therapy. The computer mediation inhibits the chance to foster *interpersonal bonds* [51] and delays the development of trust [45].

Moreover, studies show that psychologists experience discomfort in developing trust via the web and are discontent with the levels of trust achieved [1,52,53].

Digital trust requires people to start developing communication abilities *to convey empathy and attention* [54], thereby establishing a *digital empathy* to increase trust [54-56]. Developing trust via VCP is difficult as users have limited eye contact and physical interaction, which interferes with *real-time feedback* [54,57].

The following section further elaborates on eye contact and body language, which function on many levels in the psychologist-patient relationship and communication.

### Eye Contact and Body Language

The lack of eye contact [58,59] and body language [60] are major limitations of VCP. With VCP, it is difficult to detect the direction in which people are looking, and users need to rely on nonvisual pointing strategies to achieve common spatial orientation. Hence, for the practice known as *referential mapping*, users point to areas of their environments to localize specific areas in coparticipants’ remote environments [61]. This practice provides meaning to gaze and gestures when participants cannot direct their gaze to a common object and eye contact is unachievable. It has been shown that eye contact is sustained when the gaze angle (between the user and the videoconference system unit) is lower than 7° [62,63], and in VCP, there is a deviance between the user’s gaze and the camera [64]. This deviance affects the development of empathy owing to its impact on visual information [65]. Attenuation of the deviation should lead to improved therapeutic experience [64].

Eye contact is a strong form of nonverbal communication, which helps both sides to understand each other’s thoughts, intentions, and emotions [1]. Communication requires harmony between gaze, oral language, and body language [66].

Regarding body language, some challenges arise when considering the upper body and microexpressions (ie, subtle, involuntary facial expressions). Some studies emphasize the type of camera frame as important for the amount of information it conveys. Studies refer to frames including the upper body as an empathy facilitator compared with the head-only frames [60].

Micro–facial expressions are an involuntary way to express and communicate emotions [26]. However, this information is difficult to perceive via VCP. Microexpressions do not usually last >1/25 of a second [67-69]. The recognition of microexpressions is a yet developing area, but a study [67] validated and implemented a system called Video-Based Emotions Analysis System, with the prospective to distinguish basic emotions.

The emotion detection field is increasingly attracting video game researchers. The field’s high-level technology enabled video game players to share facial expressions, body language clues, and physiological indicators such as the heart beat in real time [70]. In a study conducted by Andreu-Perez et al [71], researchers were able to classify gamers’ expertise by deciphering their emotions and brain information relying on their facial expressions. This means that advanced technology implemented in specific fields can possibly be adopted for multidisciplinary purposes, such as enhancing the VCP field.

Thus, body language and the abovementioned elements are essential for smooth communication between psychologists and patients and to regulate the dynamics that TR involves. These elements are critical in developing therapeutic processes and are partially lost in the transition from in-person therapy to VCP. Hence, it becomes crucial to traverse the convolution of the VCP system by applying the Chaos Theory lenses to the psychologist-patient therapeutic processes. Our study was extensively supported by this theory because it helped us to look at the VCP as a system in which small technical alterations can generate important variations in the entire VCP structure.

### VCP and the Butterfly Effect of the Chaos Theory

By considering the complexity of VCP, we relied on the Chaos Theory to guide our exploration with psychologists. Our purpose was to understand the processes involved and the possible technical needs that may be used to enhance psychologists’ experience.

The assumption of the Chaos Theory lays on the fact that “systems are sensitive to initial conditions: any small alteration in the initial condition can produce considerable changes in the whole system” [26]. This theory is also well known as *butterfly effect*: the flap of butterfly wings generates changes in the air that can lead to the formation of hurricanes in the future. Lorenz [72,73] was able to identify and map these small changes in the system; thus, he demonstrated that chaotic systems comply with internal order criteria. The Chaos Theory has a system approach and considers *processes*; it explores variations in the whole system, and these are dependent on original circumstances, which means that the entire system is substantially *sensitive to the condition it starts with* [74].

Following this perspective, we built interviews to study which processes involved in VCP influenced psychologists’ reluctance toward this medium. More specifically, although the literature juxtaposes patients’ satisfaction with psychologists’ skepticism, little is known about the processes leading to psychologists’ diffidence toward VCP. By investigating the processes affecting VCP and the technological means supporting them, we intended to provide new important directions in terms of system behavior.

The study of the processes may arouse specific expectations regarding VCP system activities. However, minor alterations within this sensitive system might impact psychologists’ and patients’ expectations toward the VCP experience. Hence, we applied the Chaos Theory to explore with psychologists how the alteration of a factor might impact further VCP elements. Therefore, we started to use the IPO model to map the *processes* impacted by specific *inputs* (alterations) and those that may provide desirable expectations (output) regarding VCP system behaviors.

The concept underpinning the IPO model is the complexity of behaviors, interpreted as systems living in other contexts and interacting with people, machineries, and environments. This model can be used in conditions where a *process* is able to translate a certain *input* into *output* [75], and this model appears to fit well with the VCP complex system.

## Methods

### Overview

To obtain a deep comprehension of psychologists’ videoconferencing experience, we developed a qualitative research study. On the basis of the literature and our research questions, we created a semistructured interview (Textbox 1) to be presented to our sample.

We conducted these interviews in 2020 and 2021, during the COVID-19 pandemic. Owing to the pandemic, videoconference was also used for the local professionals’ interviews. The interviewer applied an empathetic and nonjudgmental approach [76].

**Sample interview questions for psychologists.**

- Could you please tell me how you set up the Video Conference (VC) with your clients? Is it easy or hard to do, and why?
- Could you please tell me what the benefits are of using VC?
- Considering that you are not in the same room with your clients, does that affect your way of feeling close and empathize with your clients? How?
- During your VC therapy sessions, how do you manage the absence in the treatment process?
- In terms of how you establish and maintain trust, how is the VC therapy experience different to the face-to-face experience?
- Let us dwell upon the use of specific technologies for therapeutic purposes
  With the current camera’s features, can you easily detect your clients’ facial expressions?
  Do you record your sessions? What do you use the recordings for?
  Does the Wi-Fi, Video and Audio quality impact the treatment’s efficiency? If so, how do you deal with this?
  Are there any technical issues that bother you and affect the quality of your work?
  Have you ever changed the platform you were using to something else for some reason? If so, could you please tell me the reason and the kind of feature were/are you looking for?
- In relation to your VCP experience, could you compare the cognitive load and effort needed to build a productive therapeutic alliance or relationship with your clients?
- In comparison with face-to-face, has the nature of your relationship with your clients changed in VC Psychotherapy, and how so?

We conducted the thematic analysis by following the recommendations by Braun and Clarke [77], which include the following: familiarizing with data, developing codes, searching, reviewing data again, and refining themes. This methodology allows the researcher to identify patterns across the information provided through a deep interpretation of the data [78]. Thematic analysis captures important information and offers comprehensive and multifaceted data interpretation. It explores experiences providing meaningful outcomes, as it does not depend on *quantifiable measures* [77].

### Ethics Approval

This study has been approved by the Psychology Health and Applied Sciences Human Ethics Committee (1955531.1) of the University of Melbourne (Australia) on February 7, 2020.

### Informed Consent

The researcher provided volunteer participants with consent forms before all the interviews, assuring their anonymity. Participants were informed that the focus of the interviews would be on their perspectives on technology and that no personal information would be collected. Furthermore, they were informed about the data collection process and their rights by the researcher through the plain language statement.

### Recruitment

After obtaining ethics approval, we started the recruitment process. Owing to the pandemic, all the interviews were conducted via videoconference platforms. Participants were recruited from different countries with the goal to obtain a heterogenous sample. Psychologists across the world were contacted directly by the PhD scholar (who is also a clinical psychologist) via LinkedIn, professional practice websites, and Official Psychologists’ Associations. They were asked via email to read the plain language statement and sign the consent form before scheduling the videoconference meeting for the interview. In total, we interviewed 17 female psychologists (having a similar educational background) across the world (n=7, 41% from Australia; n=1, 6% from England; n=5, 29% from Italy; n=1, 6% from Mexico; n=1, 6% from Spain; and n=2, 12% from the United States) who varied in their level of VCP experience. The main languages used to conduct our interviews were English and Italian (owing to the researcher’s language skills); however, all the interviews were first audio recorded and then transcribed in English.

### Data Analysis

Our qualitative analysis aimed to understand the complexity of human behavior in the VCP context. We used the thematic approach to determine new ideas and allow new themes to emerge (refer to the *Results* section) [79]. This helps researchers to better understand the reality with the involvement of participants in assessing the representation of their experience [80].

We applied the Chaos Theory lenses to explore psychologists’ behaviors in VCP. Hence, we considered VCP as a system and studied the alterations in VCP course when a change is produced. We were interested in a better understanding of how one aspect of VCP affects others.

**Figure 1:**
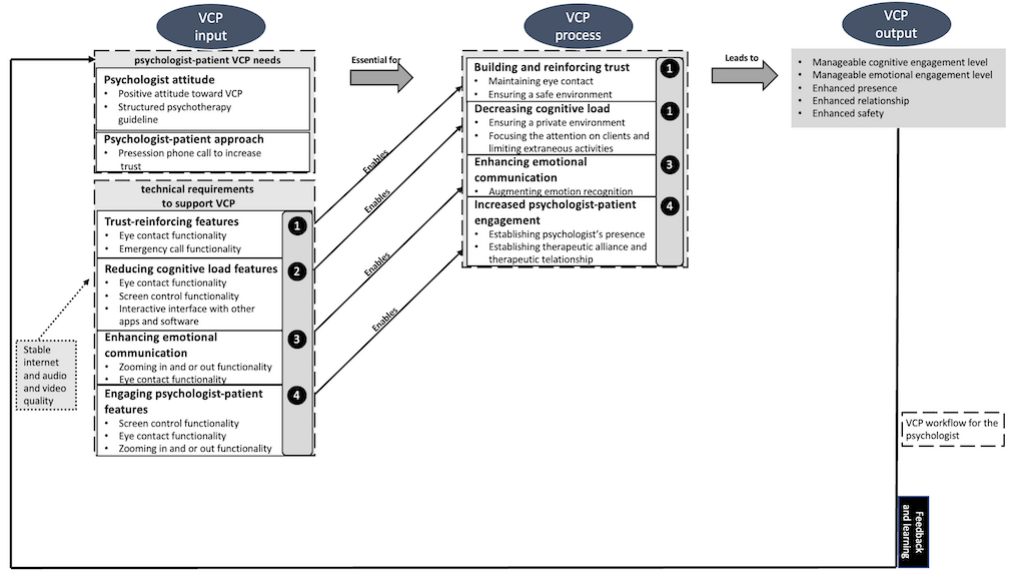
Input-Process-Output model—integration of technical features in videoconference psychotherapy (VCP).

This theory is reflected in the adoption of the IPO model (Figure 1), which is process oriented and helps us to understand what digital affordances may be necessary along each step of the VCP process.

This model is usually used in team organizations, and it shows an inactive linear sequence. Owing to this inactivity sequence, the feedback, which connects the VCP output to the VCP input, conveys the dynamic movements.

In Textbox 2, we have reported our interview results in the *Inputs and Processes* sections. We adopted the VCP-IPO model to guide us in the complexity of the system by mapping the alterations (*inputs*), *processes* affected, and desirable anticipated *outcomes.* In this environment, the VCP-IPO model underpins the interdependent set of multipurpose and multilevel features to boost psychological mechanisms that were previously missed or limited.

**Themes and subthemes based on the psychologists’ experiences of videoconferencing psychotherapy.**

**Themes and subthemes related to input considerations**

1. Input—psychologists’ attitude
  Positive attitude toward videoconference psychotherapy
  Structured psychotherapy guidelines
2. Input—psychologist-patient approach
  Phone call before the initial session to increase trust
3. Input—trust-reinforcing features
  Eye contact functionality
  Emergency call functionality
4. Input—reducing cognitive load
  Eye contact functionality
  Screen control functionality
  Interactive interface with other apps and software
5. Input—enhancing emotional communication
  Zooming in and out functionality
  Eye contact functionality
6. Input—engaging features between psychologists and patients
  Screen control functionality
  Eye contact functionality

**Themes and subthemes related to process considerations**

1. Process—building and reinforcing trust
  Maintaining eye contact
  Ensuring a safe environment
2. Process—decreasing cognitive load
  Ensuring a private environment
  Focusing the attention on patients and limiting extraneous activities
3. Process—enhancing emotional communication
  Augmenting emotion recognition
4. Process—increased psychologist-patient engagement
  Establishing psychologists’ presence
  Establishing therapeutic alliance and therapeutic relationship

## Results

### Overview

A total of 17 psychologists across the world were contacted for a semistructured interview. The analysis of these interviews resulted in the formulation of 9 themes and 19 subthemes (Textbox 2). Findings are given by reporting extracts of the interviews that were relevant for understanding participants’ personal VCP experience. Our results capture the range of digital affordances and what psychologists told us concerning their needs as part of their practice. We present our findings under the IPO phases.

As discussed in the *Introduction* section, we used the IPO model to delineate additional information about the technology and its possible involvement in therapeutic processes.

Textbox 2 illustrates our themes as inputs and processes, and it anticipates the description of inputs and processes provided by the IPO diagram.

As indicated in Textbox 2, the subthemes are reported by presenting the number of respondents. All of our participants (17/17, 100%) were lacking technology knowledge and competencies owing to their educational background; however, they provided us with important insights. Thus, although only 5 of the subthemes were agreed on as important by >50% (9/17) of the respondents, we also report the less common subthemes, as these were also consistent with the overall responses.

In the following section, we categorized our interview results into input themes and subthemes. We included verbatim quotations as evidence for our data interpretation and deep understanding.

### Input—Psychologists’ Attitude

As shown in Textbox 2, psychologists realize that attitude is important, and it can be conveyed by trust; they try to compensate for what they are missing with what videoconference has to offer. In addition, they reported the necessity to have a structured therapy, which would help them to stay on track with their clients and vice versa.

#### Positive Attitude Toward VCP

Respondents seem to underline a slow change in psychologists’ attitude; for some psychologists, the differences from FTF psychotherapy cannot be ignored, whereas other psychologists believe that it is important to engage positively with the different VCP information:

So, rather than focusing on the deprivation compared to face-to-face, I see the novelty that might be added.

#### Structured Psychotherapy Guidelines

Psychologists encourage the adoption of a structured therapy. They need controlled strategies to stay in control of the session in case of unplanned events. Web-based therapy appears to be seen as less manageable than FTF therapy, because it offers a wide range of unpredictability. Psychologists believe the following:

...It requires more effort and needs more deliberate structure as normally you would walk into a room together, which is quite symbolic.

### Input—Psychologist-Patient Approach

#### Overview

Psychologists provide some insights regarding their personal approach to VCP. They believe that it is important to start building trust with their patients before the first VCP session. Some of our respondents suggest to have a phone call before the first session; this would help in their trust building process.

#### Phone Call Before the Initial Session to Increase Trust

Psychologists are concerned about the establishment and development of trust via video. They are aware that trust is always the first milestone in therapy, in particular when the treatment is delivered via the web.

A phone call before the first video session was suggested as a strategy to establish trust:

It’s really important. It’s the highest priority. The highest, to build trust in that way. So, I very rarely see anybody online, until I have had first a call with him. A call of 45 minutes at least. So, I’ve already built credibility and trust.

Another respondent suggested to focus on improving trust from the beginning, with a presentation of the psychologist that shows credibility:

I’ve to introduce myself to him first [patient]. I consider that to be important. My presentation is pretty structured: there’re the personal aspects, the life aspects, the professional aspects...Then, with respect to the trust process, this very starting point characterizes the very process in a specific way.

### Input—Trust-Reinforcing Features

#### Overview

Trust is an essential element in the relationship between psychologists and patients, which can help the latter to feel safe [48]. To develop trust, it is necessary to be responsive to patients’ feelings and understand “what is going on.”

Psychologists feel that they are less receptive and responsive while working via video:

I think I do check maybe a bit more in Videoconferencing.

As we mentioned previously, sight restraint impedes the *real-time feedback* [54,57], and psychologists feel that having the chance to establish eye contact via video with patients might provide emotional feedback.

Here, trust also refers to psychologists’ concerns around telehealth platforms. Psychologists often avoid working via video with patients exhibiting possible harmful behaviors. They feel that patients’ safety may be compromised:

During online therapy, I would deal with this kind of situations with the greatest tension, and just the fact of not being able to deal with them at such a virtual distance; if in person, you could even physically assist the patient.

#### Eye Contact Functionality

According to our sample of psychologists, eye contact is decisive for establishing contact and trust:

One of the important things that quite happens a lot, is that my clients can’t see me when I look at them. That actually is a form of contact...You know, the experience of being seen. That physical experience is very important, and at the moment doesn’t seem to. They trust on if I look at them.

#### Emergency Call Functionality

Psychologists are concerned about initiating web-based therapies with patients exhibiting suicidal instincts or experiencing panic attacks. In such emergencies, they feel that working remotely may undermine prompt and effective support. They declare their need for an emergency call feature to connect them to hospitals and social services if something happens with their patients:

If there was a feature like that, it would be useful, of course. You know...they have a panic button that they would press and then police would be contacted. If a feature like that was done, a feature like that for the devices, it would help of course facing the – you know, being able to help the client.

### Input—Reducing Cognitive Load

#### Overview

The interviewed psychologists talk about the cognitive difficulty of having sequences of VCP sessions, with only brief time away from the screen in between. They report that VCP requires more substantial cognitive load compared with that required for FTF sessions. Most difficulties relate to the level of attention required to recognize all the nonverbal messages included in the communication. Respondents also report increased effort in reassuring patients about the psychologist’s presence.

#### Eye Contact Functionality

Eye contact was one of the most recurring topics in our interviews. According to our respondents, reduction in fatigue would enhance psychologists’ presence:

Well, I think the only way that you could do it is try, trying to keep a lot of eye-contact with them or try to look right in the camera and make sure that they feel listened to, and I think I, we psychologists have to make a huge effort, you have to be, you’ve to make a big effort to be present and to I guess, respond to them, so that they can feel like, you know, you’re there, even though you are not there like. I mean, it’s harder as work, a little bit more.

#### Screen Control Functionality

In our sample, psychologists working via the web require a high level of continuous attention. They need to consistently monitor patients’ attention, as notifications from the computer (eg, to later sessions or emails) may interfere with the outcome of the session. A respondent pointed out the following:

I want to see unit...like, an integrated unit with the remote [laughs]. That is adjustable, steady and reliable and has features that ensure confidentiality and control. That’s what I would be happy to utilize in a future.

#### Interactive Interface With Other Apps and Software

Platforms prove to be overly “rigid” for our psychologists. They would prefer platforms that optimize the VCP processes with their patients, as the therapy tends to slow down owing to the platforms’ rigidity. They claim their need to have more interactive platforms that allow them and their patients to access apps and other software without affecting the session’s flow:

But I think yes, it does. It is harder to get through the content over video-link than it is Face-to-Face. I’m finding that, because my patents do a lot of homework: they have to bring that in, so that we can work through that in sessions; however, it’s very hard for them to bring them in, so they have to email to me, and I need to read it, because the platform that I’m using doesn’t - oh, it would be so good if I had like an App that actually connected to it, where people could enter. So, I’d ask people to monitor their team behaviours, but I need to see that, so they can take it through it the next session...so, it would be really good if the platform had an App that would connect, so I could have them on a side of the camera and then their work at the other side of the screen...So, I’m sure there’s a technologic solution, an App that connects to the platforms and that people can use...but that’s sort of an issue that slows things down.

### Input—Enhancing Emotional Communication

#### Overview

According to our interviews, VCP intensity is not as relevant as the intensity of FTF therapy. This would affect the emotional connection between psychologists and patients. Thus, the building of an effective TR would be undermined:

I think the work with clients with VCP is more superficial. It’s harder to really connect with clients. I feel some of them “hold back,” as they also don’t feel the same rapport experienced in f2f sessions.

#### Zooming In and Out Functionality

The surveyed psychologists wish for zooming in (to focus on clients’ facial expressions) and out (to capture the whole body) functions. This would help to discern patients’ micro–facial expressions and body language. The function has already been used by a respondent, who said the following:

...I’ve never experienced a family call conference, better to say a therapy with several people – I’ve experienced this modality [the interviewed was a supervisor working for an institution]...and then the “in” and “out” camera usage has been actually very useful. I shall add though – the responsible for the camera activation was behind the mirror, not inside the room. Using it alone, would be like being properly in the same room. If I had the opportunity to work with a whole family, the “in” and “out” functions would surely be very useful, even if I’d to be the one to activate them.

#### Eye Contact Functionality

The interviewed professionals believe that the use of computers for psychotherapeutic purposes presents challenges to empathy conveyance and eye contact. They perceive that the computer diverts users’ attention from introspection, as users tend to monitor the medium’s performance rather than focusing on emotional aspects. This leads VCP participants to use their cognition and hinders the chances for the transmission of empathic feelings:

Using a computer makes it harder to look inwards as it necessitates looking into a screen; this often means staying with cognitions rather than focus on emotions.

### Input—Engaging Features Between Psychologists and Patients

#### Overview

Psychologists underline some alterations in the communication and relationship with their patients. A layer of psychologist-patient communication seems to be absent in videoconference sessions. Such element is usually present in the embodied communication and appears to affect a fully mutual understanding*:*

The experience of actually being in the same room with somebody. It is an experiential component that I think it’s quite important.

Psychologists would like to feel more present in the VCP experience. As mentioned previously, physical presence is important for the development of the relationship [29-31]. As videoconference precludes psychologists from physically participating in the session, professionals may increase their presence with the chance to “influence” patients’ screen.

#### Screen Control Functionality

Respondents suggested a screen control feature to restore the power dynamics disrupted by VCP. Physical absence seems to mitigate psychologists’ power dynamics and leads to changes in the psychologist-patient relationship. A psychologist clarified the following:

I can ask questions, trying to design things that are engaging to keep their attention, but sometimes I just lose them. They are just sitting in there, and they are just pretending to listen, but they aren’t listening...I would love if I could freeze them into Google Meet, so that they couldn’t go onto videogames and check their email.

Thus, the screen control feature is expected to restore psychologists’ influence in the relationship.

#### Eye Contact Functionality

Respondents require technologies that enable effective eye contact. They report that the screen diverts glances from the camera. This may hinder the establishment of a plausible eye contact. A surveyed psychologist argued the following:

I don’t know it’d be possible at a technological level but being able to really look in each other’s eyes. I mean, it’s inevitable that both I and the patient just look at the screen, and nobody happens to look at the camera. But if we do, we do not perceive each other’s glance, we only perceive the camera, as I was saying. So, there’s no moment of effective eye-contact, as happens all the time in face-to-face. Then, if it was possible –– I don’t know how, though – being able to look in the eyes umm as in face-to-face.

Our sample of psychologists shows a noticeable change in their VCP approach. However, we also gathered their concerns about trust establishment, safety, and increased cognitive load. In addition, our sample of psychologists is also distressed about the lack of eye contact, physical experience, and control in their VCP sessions and relationships with their patients.

Moreover, they believe that their VCP sessions were less emotionally intense than their FTF therapies.

In our interviews, psychologists suggested some interesting technical features that may help them in their therapy sessions:

1. Eye contact functionality
2. Emergency call functionality
3. Screen control functionality
4. Interactive interface with other apps and software
5. Zooming in and out functionality

In the following section, we grouped our interview results into 4 process themes and subthemes, including, as mentioned previously, verbatim extracts as an indication for our data interpretation and comprehension.

### Process—Building and Reinforcing Trust

#### Overview

Psychologists recognize that eye contact plays a key role in establishing trust. According to our sample, trust would be improved by maintaining eye contact during sessions. It would also be important to ensure a safe VCP environment because the distance is perceived as an insuperable impediment. Psychologists are reluctant to assist patients with severe mental illness via video.

#### Maintaining Eye Contact

According to our sample, maintaining eye contact via video would be important to establish trust and emotional understanding. Wilson et al [47] pointed out that impediments to vision affect the development of trust, as the opportunity to identify joint goals is impaired.

On the basis of literature, gaze helps psychologists and patients to establish trust [47]. The impossibility of establishing a mutual gaze seems to bother all the surveyed professionals (17/17, 100%).

As chances for eye contact are scarce, psychologists fear that patients may not perceive their empathy. Appropriate levels of eye contact may reassure patients that they are being listened to. Therefore, insufficient levels of eye contact may hinder trust reinforcement:

Yes. I feel like is hard because they feel more distance so, in order to build the relationship, you have to be more present, you have to really make eye-contact, try to give them something – just to say “no” or “yes” after, just to make them know that you are there, that you are listening, right?

Eye contact functionality (refer to Textbox 2—input 3) would allow better trust reinforcement, as trust would be improved by maintaining eye contact during sessions.

#### Ensuring a Safe Environment

Psychologists express their concerns in dealing via VCP with patients with severe mental health issues. They feel that their work is facing some limitations, as working remotely hinders patients’ assistance in case of emergencies, critical incidents, or emotional and behavioral escalations. Therefore, psychologists appear to be more careful in avoiding reactions they could not control remotely:

You have less control, because you know, if the client is a suicidal, and if there’s an immediate risk in a face-to-face situation, you can contain that in the room, and then ask for an ambulance, and they come in and drop the client. However, when you have this Videoconferencing situation, first of all the client is connecting with you from home, so you have to make sure – if there are any risk issues, you can call the police, but you can’t hold the client. Does that make sense?

The emergency call functionality mentioned in the *Input—Trust-Reinforcing Features* section (refer to Textbox 2—input 3) would favor trust reinforcement by ensuring that both users have a safe environment to interact in. This would enhance the users’ confidence in the medium. With this functionality, psychologists would feel more confident and less apprehensive in case of patients’ emotional escalations (panic attacks, suicidal thoughts, high level of anxiety, etc).

### Process—Decreasing Cognitive Load

#### Overview

Psychologists’ overload is owing to the greater focus that VCP demands. Although they are still expected to provide attentive active listening and avoid distractions, psychologists may need to reassure patients about their attention despite technical interruptions. There is a component of video fatigue that refers to an exhausting sensation [81] caused by an increased level of attention, experienced after videoconferencing [82]. The functionalities reported in the *Input—Reducing Cognitive Load* section would help psychologists to decrease their load and fatigue and ensure a private environment, focusing the attention on patients and limiting extraneous activities.

#### Ensuring a Private Environment

Possible technical interruptions affect the private environment that psychologists try to offer to their patients. This means that psychologists need “to repair the situation by making sure the patient feels listened to in spite of the interruption.”

Thus, the input functionalities suggested (refer to Textbox 2—input 4) may help psychologists and patients to ensure a private web space.

#### Focusing the Attention on Patients and Limiting Extraneous Activities

According to our sample, patients are often engaged in activities other than therapy during VCP sessions. Consequently, psychologists feel that they strive to engage with their patients effectively:

The cognitive load is more intense, as I find it takes increased concentration to stay on track and avoid distractions.

The set of technology features suggested and reported in Textbox 2—input 4 may help psychologists in decreasing their cognitive load.

### Process—Enhancing Emotional Communication

#### Overview

Psychologists have the feeling that working with patients via video is superficial and it is difficult to bond with them. Furthermore, they usually need to ask confirmation for possible emotional reactions so as not to misinterpret patients’ emotional state. Moreover, psychologists seem to underline a sort of verbal and emotional communicative misunderstanding based on some unclear aspects that are not readable through video. Psychologists declared that they focus more on the communicative aspects of the therapy “replacing” their physical absence:

I think I have to work harder at picking up non-verbal clues. I think I have also to work harder at communicating things that are cut at communicating things verbally, at communicating nonverbally as in a room. So, for example, a compassionate word, just resting my eyes on the client so to show that I’m caring: these things need to be verbalized and specifically communicated.

#### Augmenting Emotion Recognition

Patients expect to feel emotionally comprehended. According to our interviews, psychologists often struggle to recognize their patients’ emotions through VCP. Thus, psychologists need to ask more questions to patients to clarify the emotions that emerged during sessions. For instance, a respondent stated the following:

...However, because I can’t see all their whole-body language, I can’t tell how much they are distressed, or I can’t ask them the right questions in the right moment. That makes all much harder. So, I have to ask “Can you tell me if you’re getting distressed? I can’t see that across the video-link as effectively.”

The features advised by our sample, such as zooming in and out and eye contact functionality (refer to Textbox 2—input 5), may help them to enhance their emotional understanding and communication.

### Process—Increased Psychologist-Patient Engagements

#### Overview

The technologies included in the input—engaging features, such as screen control, eye contact, and zooming in and out functionality (refer to Textbox 2—input 6), aim to help psychologists and patients to engage in telehealth sessions. According to our sample, these features would contribute to improve the communication and relationship between psychologists and patients by enhancing their presence in the session. Consequently, engagement may also be enhanced by the establishment of TA and TR. These elements would help psychologists to have a better control over the relationship.

#### Establishing Psychologists’ Presence

The relationship between psychologists and patients is a crucial aspect of the therapy. On the basis of our interviews, psychologists claim that the power dynamics imply change in VCP because of the absence of their physical presence:

The nature of the relationship also changes in terms of power dynamics. The client has more power. I had clients that have decided to make a cup of tea, answer the door or take a break whereas in a therapy room this is usually not possible.

#### Establishing TA and TR

Psychologists find that working on the TA and TR remotely is more arduous than that through FTF therapy. Regarding TA, a psychologist argued the following:

The distinction with a new client is that the Alliance I think has a greater challenge...So, I would say that’s significantly more, and I’m exhausted by it.

Regarding TR, another psychologist stated the following:

...I think it requires a little extra-effort on the psychologist’s heart, to put it on the table, to say “this is weird,” “how do you feel about that?” “how I feel about that?” “let’s monitor that.” Whereas I think that in person, we just take for granted that we can build the relationship by being in the face-to-face.

After presenting our input and process themes and subthemes, it is relevant to offer our psychologists’ responses about the technical quality. They claim the importance of audio, video, and Wi-Fi stability, which seems to be crucial in supporting the flow of their sessions.

### Technology Quality

Psychologists talk about the importance of being supported by stable audio, video, and Wi-Fi to maintain communication flow. Although the enhancement of both audio and video quality appears to be desirable, results show that improved audio is usually preferred to better video quality.

### Stable Internet and Audio and Video Quality

Psychologists would like to be supported by high-quality audio and video channels. However, Wi-Fi also has huge importance, as sessions rely on its quality:

If the line has problems, the internet’s too slow or it just comes and goes, if there are problems, it definitely impacts the session. Yes.

Our interviews show that the audio is generally considered more important than the video:

If the audio is buzzing, and you can’t quite hear the emotions in your client’s voice, that’s a big problem. You can’t work, in that case. You need at least one channel that is working. I think, even if it was the visual, with the audio you would make a good work: for me, it has not to be the audio, because I suppose that our communication is primarily verbal, and there’s a lot of intoning and tone of voice, you can tell a lot of non-verbal things just by hearing a person’s tone of voice. But that could be a practice thing for me. I do not know.

Thus, audio, video, and Wi-Fi (particularly the audio channel) stability seems to be the precondition for successful communication.

This technical stability could lead psychologists and clients to start developing their relationships and enhancing the efficiency of their communication flow without experiencing broken VCP sessions.

## Discussion

### Principal Findings

The contribution of this paper lays on the identification of the processes involved in psychologists’ therapies and mostly on the identification of a technical set of features that may support psychologists to develop those processes. Psychologists’ technical requirements are meant to help them and their patients to improve and reinforce the establishment of trust, decrease the cognitive load, enhance emotional communication, and increase psychologist-patient engagement. Therefore, psychologists suggested the following set of features: eye contact functionality, emergency call functionality, screen control functionality, interactive interface with other apps and software, and zooming in and out functionality.

This may reduce psychologists’ reluctance and optimize their therapeutic work. Our study highlights psychologists’ need to enhance their control over the relationship; in VCP, they deal with elements of uncertainty [1,28,52,83].

However, research does not identify the processes involved, and it does not provide recommendations to deal with them remotely.

Figure 1 shows our VCP-IPO model that we have developed based on the results. In this figure, we present how a particular digital affordance has an impact on therapeutic aspects.

### Input—Psychologist-Patient VCP Needs

Referring to Figure 1, our earlier results underlined the importance of finding new strategies to build a foundation of trust. The COVID-19 pandemic pushed psychologists to leave their comfort zone and be flexible toward the opportunities that VCP had to offer. For example, some psychologists develop trust in their patients even before the first video encounter by making a phone call before the first session. Scheduling beforehand calls for building trust does not seem to be a common habit among psychologists. Patients and psychologists only interact directly when the therapy session begins, as the session booking usually occurs through the reception or websites. Thus, a new tendency arose, highlighting psychologists’ willingness to implement strategies to improve VCP*.* Besides showing psychologists’ flexibility toward VCP, the trend represents a new outcome compared with the data provided in the literature so far. Previous studies did not explore psychologists’ strategies in dealing with VCP, and most of the studies in the field underline psychologists’ disinclination toward VCP [23,24,27,52].

Although our interviews underline a change in psychologists’ attitude, VCP remains incredibly complex, and psychologists seek structured therapy parameters to navigate it.

For example, they would like to have founded directions to guide their work through video, as most of them were never trained in conducting VCP. According to our interviews, communication and relationship control play key roles for psychologists, who require additional features from platforms to feel properly supported in their experience.

In the following section, we will examine the technical requirements emerging from our interviews. The technical set of features introduced previously in the paper may support and enable the processes mentioned in Textbox 2.

### Input—Technical Requirements to Support VCP

Our results indicate that psychologists demand more technical control to enhance copresence in VCP sessions and strengthen their power dynamics over the relationship.

The functionalities required (eye contact functionality, emergency call functionality, screen control functionality, interactive interface with other apps and software, and zooming in and out functionality) would enable psychologists’ practice, empowering the development of therapeutic processes.

A study conducted by Kneeland et al [84] lists the technical and clinical challenges faced by psychologists in VCP sessions. The study also suggests a diverse communicational approach as a solution for those challenges. Another study led by Muir et al [85] introduces some organizational requirements such as an appointment system, safe storage for clients’ documentation, computer-generated staying room, and the chance for psychologists to decide when to start and conclude VCP sessions.

However, the literature does not provide a strong contribution in terms of which technical features would be helpful to support psychologists in their therapeutic work. Our interviews focused on psychologists’ feedback about VCP to explore how the critical issues emerging from their experiences might be addressed and the therapeutic processes might be enabled. Consequently, our participants believe that the mentioned set of features (eye contact functionality, emergency call functionality, screen control functionality, interactive interface with other apps and software, and zooming in and out functionality) may be highly helpful in overcoming the communicative and relational difficulties posed by VCP. Thus, the technologies presented may support psychologists to deal with the challenges that VCP presents to trust building, emotional understanding, cognitive load, and relationship processes.

In the following section, the impact of these features on psychologists’ processes are presented.

### VCP Processes

Figure 1 shows that the quality of the VCP processes (reinforcement of trust, reduction of the cognitive load during VCP sessions, and enhancement of emotional communication and level of engagement between psychologists and patients) would benefit from the implementation of specific technologies. The technologies mentioned in the *VCP Input* section would enable psychologists to experience reinforced trust through the introduction of the eye contact functionality and emergency call functionality (refer to VCP input—process box 1 in Figure 1). The precondition that allows the development of the stated processes is an increased verbal and nonverbal communication understanding. In our interviews, important data emerged regarding psychologists’ communication, which tend to be more explicative and direct. This is because psychologists strive to share and decode emotions and thoughts as they are interacting via video. The challenge stems from the difficulty to convey and receive abstract information as there are not sufficient visual and contextual clues to fully comprehend the interaction mode.

Psychologists rely on strategies of abstract communication, such as metaphors, to verbalize *abstract emotional states* without *stressing* the patient [86]. Through the eye contact functionality (refer to VCP input—process box 1 in Figure 1), it may be possible to enhance this communication control because it may allow psychologists to provide *real-time feedback* [54,57]. This would operate as a multipurpose functionality; it may help to *control* *communication* among individuals. This is a quality of the embodied communication that is called metacommunication [87].

Moreover, the features in box 2 (refer to VCP input—process box 2 in Figure 1) might enable psychologists to increase their trust not only in their patients but also in telehealth platforms. The control mentioned in the *Input—Psychologist-Patient* *VCP Needs* section (refer to VCP input in Figure 1) is also reflected in the screen control feature (refer to VCP input—process box 2 in Figure 1) as psychologists would be able to have an impact on patients’ environment. Consequently, the presence may be enhanced.

Moreover, by limiting their patients’ distractions and focusing more on therapy, psychologists could provide a private environment. This, along with the interactive interface with other apps and software (refer to VCP input—process box 2 in Figure 1), may be echoed in their cognitive load. Psychologists would be able to maximize their attention, communication, and emotional energy.

Furthermore, the introduction of the zooming in and out function in box 3 (refer to VCP input—process box 3 in Figure 1) may reduce the emotional energy. This functionality would permit psychologists to shift from head-only frame to extended-body frame to detect micro–facial expressions and more contextual clues. In this way, psychologists could be more responsive to and connected with their patients.

As shown in box 4 (refer to VCP input—process box 4 in Figure 1), one of the major impacts of the adoption of these technologies may be the high level of engagement between psychologists and clients. These features could enhance psychologists’ presence and control in the relationship. In addition, the power dynamics between psychologists and clients may benefit from the features’ implementation, as psychologists would be provided with (the functionality to achieve) major control of the patients’ screen. Thus, it would be possible to build TA and TR.

The introduction of the technical set of features to support videoconferencing psychotherapy (VCP input in Figure 1) and their potential impact on psychologists’ processes (VCP processes in Figure 1) may lead psychologists to the desired outcome (VCP output in Figure 1) presented in the following section.

### VCP Output

The abovementioned technical set of requirements (refer to VCP input boxes 1-4 in Figure 1) might lead psychologists to be more in control of their therapies and relationship dynamics. The lack of control affects psychologists’ experience, and it is mostly perceived as a huge impediment that affects TR. Control refers to the situation in which *each participant is able to control or constrain the behavior of the other* [88]. Consequently, therapies might be more cognitively and emotionally manageable than before, and psychologists would not be required to puzzle mental pictures about *what is going on* in the session owing to *elements of uncertainty* [28].

Previous studies [26,89] mentioned that the lack of physical presence affects psychologists’ work owing to missing information that is only detectable in person. Nevertheless, our study demonstrates that these missing cues may be recovered through the introduction of the abovementioned features. This could be beneficial for enhancing the psychologist-patient relationship.

Furthermore, the emergency call functionality may guarantee a safe VCP experience for psychologists and patients. Psychologists’ misgivings about treating given types of patients via the web [52] would be addressed. Thus, they may be inclined to treat patients with severe mental illness via video.

Figure 1 shows the connection between the VCP output and the VCP input. This connection states that psychologists are provided with feedback, which is the information they receive about the learning outcomes. This may improve psychologists’ entire videoconference experience, which also depends on the stability of the Wi-Fi and the quality of audio and video features.

According to Figure 1, through the introduction of the set of features suggested by our respondents, it may be possible to have an impact on psychologists’ processes, leading them to experience enhanced VCP sessions. Psychologists would feel supported in their cognitive load, thus boosting their emotional engagement and augmenting their sense of presence and safety. Furthermore, they would be able to enrich their relationships with their patients.

### Limitations

The study started in February 2020, at the beginning of the COVID-19 pandemic. The pandemic had an impact on psychologists’ workloads, which overall reduced their availability to participate in our study. However, we were able to obtain interviews from 17 psychologists out of hundreds of psychologists who were contacted. We interviewed professionals from different countries, which gave us a heterogenous sample for our study.

We were not able to track, evaluate, observe, or analyze the effectiveness of each psychologists’ session. We focused exclusively on psychologists’ perceptions and perspectives.

Another limitation is the lack of follow-up with them to establish their perspectives after the pandemic; extensive use of telehealth platforms may highlight further notes or review some previous positions.

It would be intriguing to know clients’ viewpoint to examine the contrast and diverseness with psychologists.

Furthermore, our study lacks design-related insights from telehealth platform providers; we believe it is essential to involve them in the immediate future.

### Conclusions

To the best of our knowledge, this is the first study to describe psychologists’ processes involved in VCP and the technology requirements that would enhance the effectiveness of VCP from psychologists’ perspective. This study also supports our previous study [1] that conceived the computer as part of the interaction and not only as a medium through which sessions occur. All the interdependent features that emerged from our interviews tend to humanize the computer by providing it with more sensors. As with human senses, these could be used for multiple purposes during VCP sessions. Just as psychologists benefit from a full range of sensory information in FTF sessions, they require more *contact* with the computer to achieve better insight in VCP sessions.

This observed tendency to *humanize* or enrich the sensory world of the computer contributes to the general perception of it as an actual embodied part of the communication. Thus, the computer played a key role in communication, rather than remaining as a technical medium. By adopting the IPO model to analyze the VCP environment (VCP-IPO model), the relationship experience may be enhanced and the technologies to support it can be better designed. Psychologists could have more control in their VCP sessions, and the computer would play an interactive role with psychologists.

The strength of our model lies in uniting the possibilities that technology provides and the urgence of feasible strategies to conduct VCP. Thus, we combined and crossed separate disciplines such as technology and psychology to improve the experience with and effectiveness of VCP. Furthermore, this model highlights the possibilities of technology in enhancing the building of TA on VCP platforms.

Future studies should investigate the role of the computer in therapeutic treatments, not as a medium, but as a third part in the therapeutic context.

Moreover, it would be important to examine the feasibility and efficacy of these mentioned technologies involved in the psychologists’ processes. On the basis of the videoconference technologies currently adopted by psychologists and the help these aids may provide to remote people with mental health issues, it is particularly important to acknowledge and support psychologists’ technical requirements.

It is also necessary to examine psychologists’ level of training. Our participants had not received any training related to working with patients via VC, and their level of comfort with computers has not been investigated.

Further studies should also explore psychologists’ perspectives about the development of TA and TR in VCP, along with psychologists’ and patients’ degree of satisfaction with the clinical outcomes of VCP.

We hope that the VCP-IPO model presented in this study will expand, as addressing such technological requirements would facilitate psychologists’ therapeutic work and enhance the effectiveness of VCP.

## Abbreviations

FTF: face-to-face
IPO: Input-Process-Output
TA: therapeutic alliance
TR: therapeutic relationship
VCP: videoconference psychotherapy
